# The SMN Protein is a Key Regulator of Nuclear Architecture in Differentiating Neuroblastoma Cells

**DOI:** 10.1111/j.1600-0854.2009.00972.x

**Published:** 2009-09-04

**Authors:** Allyson K Clelland, Nicholas P Kinnear, Lisa Oram, Julie Burza, Judith E Sleeman

**Affiliations:** 1 School of Biology, University of St Andrews, Bute Medical BuildingsSt Andrews, Fife KY16 9TS, UK; 2 Division of Pathology and Neuroscience, University of Dundee, Ninewells Hospital and Medical SchoolDundee, DD1 9SY, UK

**Keywords:** Cajal body, differentiation, gem, nucleus, snRNP maturation, spinal muscular atrophy, survival motor neuron

## Abstract

The cell nucleus contains two closely related structures, Cajal bodies (CBs) and gems. CBs are the first site of accumulation of newly assembled splicing snRNPs (small nuclear ribonucleoproteins) following their import into the nucleus, before they form their steady-state localization in nuclear splicing speckles. Gems are the nuclear site of accumulation of survival motor neurons (SMNs), an insufficiency of which leads to the inherited neurodegenerative condition, spinal muscular atrophy (SMA). SMN is required in the cytoplasm for the addition of core, Sm, proteins to new snRNPs and is believed to accompany snRNPs to the CB. In most cell lines, gems are indistinguishable from CBs, although the structures are often separate *in vivo*. The relationship between CBs and gems is not fully understood, but there is evidence that symmetrical dimethylation of arginine residues in the CB protein coilin brings them together in HeLa cells. During neuronal differentiation of the human neuroblastoma cell line SH-SY5Y, CBs and gems increase their colocalization, mimicking changes seen during foetal development. This does not result from alterations in the methylation of coilin, but from increased levels of SMN. Expression of exogenous SMN results in an increased efficiency of snRNP transport to nuclear speckles. This suggests different mechanisms are present in different cell types and *in vivo* that may be significant for the tissue-specific pathology of SMA.

The inherited neurodegenerative disorder, spinal muscular atrophy (SMA) results from expression of too little of the protein survival motor neurons (SMNs) (1). SMA is characterized by the degeneration of motor neurons in the spinal cord, resulting in progressive atrophy of skeletal muscles. SMA occurs with a frequency of 1 in 10 000 live births, making it the leading genetic cause of infant death. There is currently no effective treatment. Although the genetic cause of the disease is known, the mechanism by which lowered SMN expression leads to selective loss of motor neurons is not understood. SMN is found in the cytoplasm of the cell and in small nuclear bodies called gems [gemini of Cajal bodies (CBs)] (2). Gems are closely related to CBs and in many cultured cell lines the two are indistinguishable. *In vivo* CBs and gems are often seen as separate structures in foetal tissues, with the percentage of nuclear bodies containing both SMN and coilin increasing with foetal age. This suggests that their colocalization may be developmentally regulated (3). Interestingly, the greatest degree of colocalization of CBs and gems, 50%, is seen in motor neurons of the spinal cord. Cytoplasmic SMN is required for the correct assembly of essential pre-mRNA splicing factors called small nuclear ribonucleoproteins (snRNPs). The core snRNPs, Sm proteins, are assembled in a ring around the core snRNA (4,5) by a process involving SMN together with a number of associated proteins [reviewed in (6)]. CBs are the first site of accumulation of newly imported snRNPs in the nucleus (7) and they have been implicated in nuclear stages of snRNP biogenesis (8,9). There is also increasing evidence that they have role in snRNP recycling (10–12). Thus, gems and CBs are linked both by their close physical association and by their involvement in the same molecular pathway.

Because pre-mRNA splicing factors are required in all cells, their biogenesis can be regarded as a housekeeping function. The total absence of SMN is lethal to cells (13). The highly selective pathology seen in SMA is, therefore, difficult to explain. There is evidence to suggest that SMN may have additional functions in neural cells, as it is found in granules actively transported in neuronal processes (14). In addition, defects in axon outgrowth have been reported in zebra fish and mouse models of SMA (15–17). However, the defects seen in zebra fish can be rescued by the injection of exogenous snRNPs (16). While motor neurons are typically regarded as the affected cell type in SMA, there is also some evidence that differentiated muscle cells may be damaged by lowered levels of SMN (18,19), while defects in motoneuron junctions have been demonstrated to be the earliest pathology in mouse models of SMN (20,21). Recently, it has been suggested that lowered levels of SMN selectively affect the biogenesis of certain snRNPs, particularly those involved in the minor spliceosome (22,23) and lead to widespread splicing defects (23). It is clear, therefore, that a fuller understanding of the mechanisms controlling snRNP biogenesis is required. In particular, any differences seen between differentiated and undifferentiated cells and, specifically, neural and non-neural cell types may shed light on the molecular pathology of SMA.

There is evidence that protein methylation is required for the correct localization and function of certain nuclear proteins (24,25). In particular, the CB signature protein, coilin, has been demonstrated to contain symmetrically dimethylated arginine (sDMA) residues. In HeLa cells, the correct methylation of coilin is required for its binding to SMN and for the colocalization of gems and CBs. Strains of HeLa showing separation of CBs and gems were demonstrated to undermethylate green fluorescent protein (GFP)-coilin (25). In this study, we have investigated the relationship between CBs and gems and the methylation of endogenous coilin in the human neuroblastoma cell line SH-SY5Y as it undergoes differentiation and neurite outgrowth. The colocalization between CBs and gems increases during differentiation, mimicking changes seen during development *in vivo*. However, despite the presence of sDMAs in coilin in SH-SY5Y cells, we find no evidence for changes in the methylation of coilin during differentiation. In contrast to previous studies in HeLa cells and fibroblasts (26,27), we find that the level of expression of SMN is a key regulator of the relationship between CBs and gems in differentiating neuroblastoma cells. As the prevailing view is that the role of the CB/gem complex is to increase the efficiency of snRNP maturation (11,28), these findings have important implications for the understanding of the tissue-specific nature of SMA.

## Results

### Differentiation of SH-SY5Y cells is associated with increased colocalization of Cajal bodies and gems

**Figure 1:**
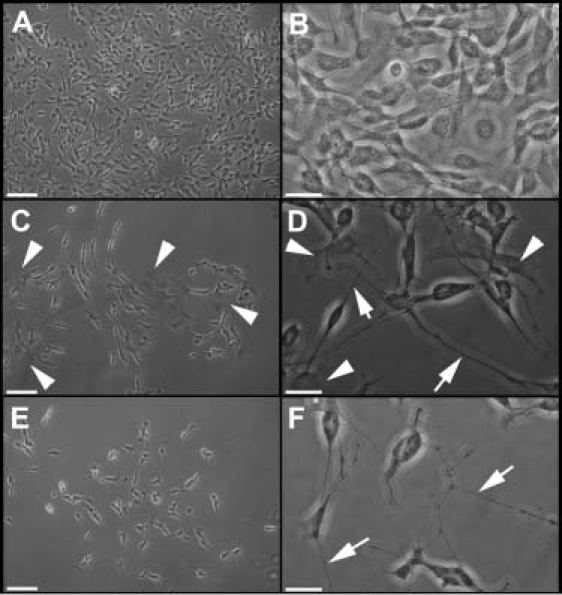
**SH-SY5Y cells can be differentiated in culture.** Phase-contrast images of SH-SY5Y cells undifferentiated (A and B) and following differentiation for 10 days with retinoic acid (C and D) or 4 days with retinoic acid followed by 6 days with BDNF (E and F). Using either differentiation protocol, the cells extend long neural processes (arrows). Using retinoic acid alone, a few cells do not adopt a neural morphology (arrowheads in C and D). Bar = 100 μm (A, C and E) or 30 μm (B, D and F).

**Figure 2:**
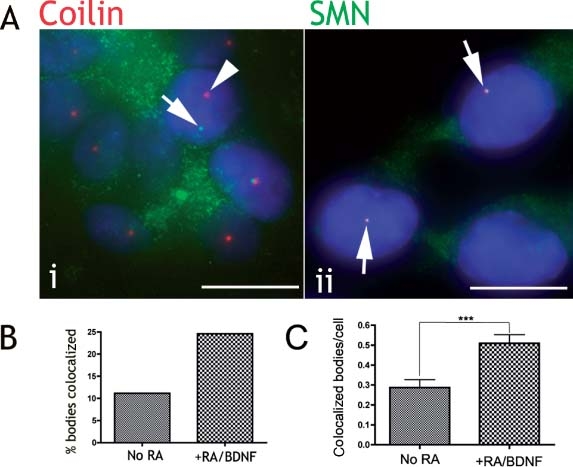
**Cajal bodies and gems increase their colocalization on differentiation.** A) SH-SY5Y cells grown without (i) or with (ii) RA followed by BDNF. Nuclear gems containing SMN alone (green, arrow in i) and CBs containing coilin alone (red, arrowhead in i) are seen frequently in undifferentiated cells. In differentiated cells, the proportion of nuclear bodies containing both SMN and coilin is increased (arrows in ii). Bar = 10 μm. B) Graph showing the percentage of total nuclear bodies that contain both SMN and coilin before and after differentiation with RA and BDNF (total *n* = 335 cells, three independent experiments). C) Graph showing the increase in number of colocalized bodies per cell. The change is statistically significant using an unpaired, two-tailed *t*-test (p < 0.001).

In many commonly studied cell lines, CBs and gems are indistinguishable from each other. CBs are identified by the presence of coilin and are implicated in nuclear stages of splicing snRNP maturation, while gems contain the SMN protein known to be important for earlier, cytoplasmic, stages of snRNP biogenesis. *In vivo*, however, CBs and gems are separate in foetal tissues and colocalized in the equivalent adult tissues (29). The neuroblastoma cell line, SH-SY5Y, can be induced to undergo differentiation *in vitro* by addition of retinoic acid (RA) (Figure 1C,D) or by the sequential use of RA followed by brain-derived neurotrophic factor (BDNF) (Figure 1E,F) (30,31). Both treatments result in the extension of neurites from the cells (Figure 1D,F, arrows). However, some cells within the population remain undifferentiated in cultures treated with RA alone (Figure 1C,D, arrowheads). These cells are not seen in cultures subsequently treated with BDNF, in which all cells develop a neural appearance providing a reproducible method to produce uniformly differentiated cells. To investigate the relationship between CBs and gems in undifferentiated and RA+BDNF-differentiated SH-SY5Y cells, cells were fixed with 3.7% paraformaldehyde and nuclear bodies detected using antibodies to the CB marker, coilin, and to SMN. In undifferentiated SH-SY5Y cells, CBs and gems are present predominantly as separate structures (Figure 2Ai), with just 10% of nuclear bodies containing both SMN and coilin (Figure 2B). Following differentiation with RA and BDNF, the percentage of nuclear bodies containing both SMN and coilin increases to around 25% (Figure 2Aii,B). There is a corresponding statistically significant increase in the number of colocalized CBs (coilin) and gems (SMN) per cell (Figure 2C). SH-SY5Y cells thus represent a suitable model of neural differentiation for examining molecular mechanisms regulating the relationship between CBs and gems. Because the RA+BDNF differentiation protocol involves several days' growth in serum-free medium, SH-SY5Y cells were grown for 10 days in serum-free medium to ensure that the changes seen were not because of growth arrest resulting from serum starvation. Serum-starved cells did not show any increase in the colocalization of nuclear bodies (data not shown).

### Global downregulation of methylation in SH-SY5Y cells leads to a separation of coilin and SMN bodies and a loss of coilin-positive bodies in general

**Figure 3:**
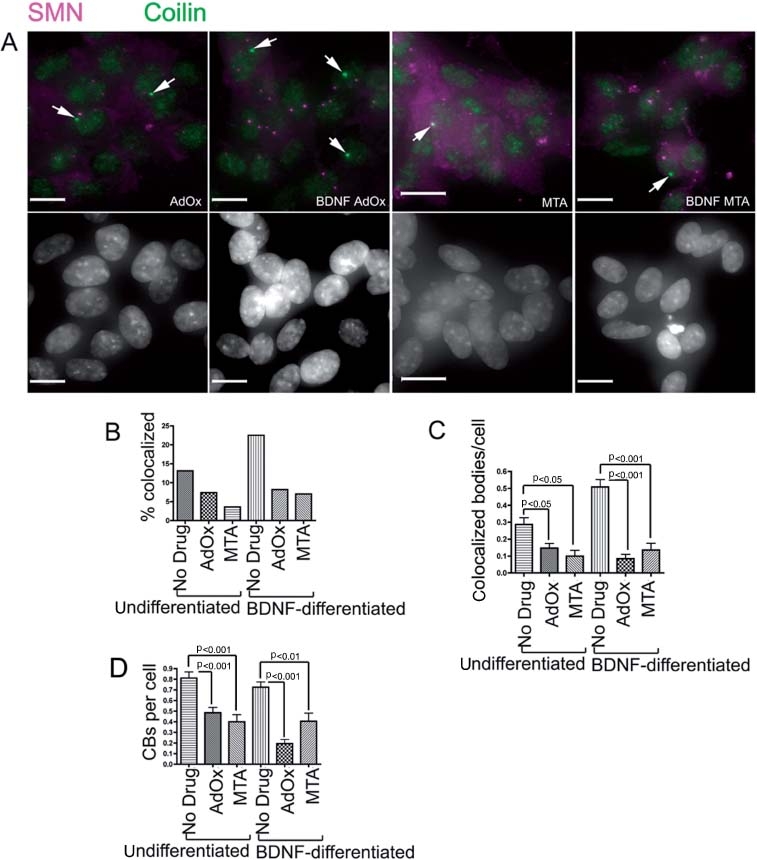
**Inhibition of protein methylation causes separation of CB and gems.** A) Top panel: antibodies to SMN (magenta) and coilin (green) show very little colocalization in undifferentiated or BDNF-differentiated cells treated for 24 h with AdOx or MTA. Very few coilin-containing CBs are seen (arrows). Bottom panel: DAPI (4′-6-Diamidino-2-phenylindole) counterstaining of nuclei. B) The percentage of nuclear bodies containing both SMN and coilin is decreased in cells treated with AdOx or MTA (total *n* = 657). C) The number of bodies per cell containing both SMN and coilin is decreased in cells treated with AdOx or MTA. D) The average number of coilin-positive foci per cell decreases on inhibition of methylation. Changes between untreated and treated populations are significant using one-way anova with Bonferroni multiple comparison post-test.

The CB marker protein, coilin, has been shown to be subject to symmetrical dimethylation of arginine (24,25) with this modification (sDMA) increasing the affinity of coilin for binding both to SMN and to the Sm protein components of splicing snRNPs in *in vitro* binding assays (32). It has thus been proposed that the presence of methylated coilin results in the formation of nuclear bodies containing both coilin and SMN, with hypomethylated coilin being present in cells with separate CBs and gems. The inhibitor of protein methylation, 5′-deoxy-5′-methylthioadenosine (MTA), has been shown to result in the appearance of gems, containing only SMN, in HeLa cells that normally contain colocalized CBs and gems (24). A different methylation inhibitor, adenosine dialdehyde (AdOx), was shown to decrease methylation of coilin, but did not result in separation of CBs and gems (25). To determine if protein methylation is important for the association between CBs and gems in SH-SY5Y cells, we treated undifferentiated and RA+BDNF-treated cells with AdOx and MTA. In contrast to results from HeLa cells, both inhibitors resulted in a decrease in colocalization of coilin and SMN (Figure 3A–C). This was accompanied by a general decrease in the number of nuclear CBs present (Figure 3D), suggesting that the decreased association between CBs and gems results from a loss of CBs, rather than a separation of colocalized bodies.

### RNAi-mediated reduction of PRMT5 affects HeLa cells and SH-SY5Y cells differently

**Figure 4:**
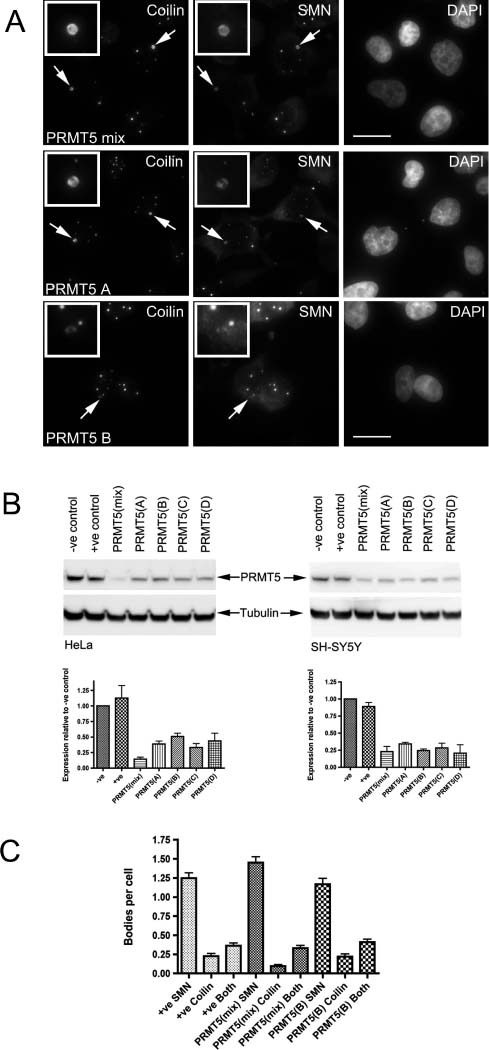
**Reduction of PRMT5 by RNAi results in abnormal nuclear foci containing SMN and coilin in HeLa cells, but not in SH-SY5Y cells.** A) HeLa cells electroporated with PRMT5 siRNA duplexes for 48 h labelled with antibodies to SMN and coilin and counterstained with DAPI. Both antigens are found in abnormal nuclear foci (arrows and insets), whether the duplexes are used as a mix (top row) or separately. Bar = 10 μm. B) Immunoblots to confirm the efficiency of depletion of PRMT5 from HeLa and SH-SY5Y cells. Graphs represent pooled data from three experiments, normalized to tubulin signal and plotted as a proportion of the negative control. C) SH-SY5Y cells with lowered PRMT5 show no significant changes in the distribution of coilin and SMN in nuclear bodies (one-way anova with Bonferroni multiple comparison post-test, total *n* = 632, p > 0.05 for all relevant comparisons).

The metabolic inhibitors Adox and MTA cause a general inhibition of protein methylation. To focus further on the role of methylation of coilin in maintaining CB integrity, we employed siRNA to reduce the levels of the methylase protein methyl arginine transferase 5 (PRMT5), which is responsible for addition of sDMA modifications to coilin and the Sm proteins (24,33). Four duplexes were used individually and as a mix. Hela cells electroporated with duplexes to reduce the levels of PRMT5 (Figure 4A,B, and other data not shown) showed a striking reorganization of coilin into large irregular structures (arrows). These structures were seen in 46% of cells electroporated with a mix of four duplexes (*n* = 126), 17% with duplex A (*n* = 233) and 26% with duplex B (*n* = 156). This compares with less than 1% in control cells (*n* = 468). Interestingly, the subcellular distribution of SMN was also disrupted in these cells, with colocalization of SMN and coilin seen in the aberrant irregular structures. This suggests that direct molecular interaction between methylated coilin and SMN is not the major cause of colocalization of CBs and gems. Electroporation of SH-SY5Y cells with siRNA duplexes to reduce PRMT5 levels did not result in the presence of abnormal nuclear bodies or major changes in the distribution of coilin and SMN between nuclear bodies (Figure 4B,C, and other data not shown). This adds further weight to the suggestion that nuclear bodies are regulated differently in different cell types.

### Coilin shows equivalent methylation and affinity for binding SMN in undifferentiated and differentiated cells

**Figure 5:**
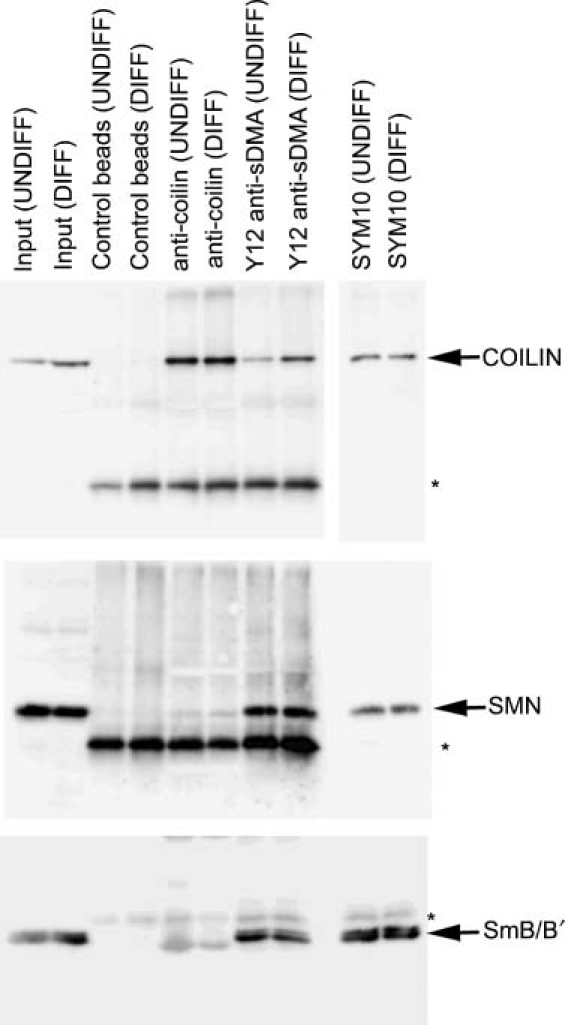
**Anti-sDMA antibodies immunoprecipitate coilin, SMN and Sm proteins from undifferentiated and differentiated SH-SY5Y cells.** Equal amounts of whole cell lysates from undifferentiated or BDNF-differentiated SHSY-5Y cells were used for immunoprecipitation using anti-coilin antibodies and the anti-sDMA antibodies Y12 and SYM10. Duplicate blots of immunoprecipitated material were probed with anti-coilin (top), anti-SMN (middle) and Y12 anti-sDMA (bottom). Endogenous coilin was immunoprecipitated by anti-coilin, Y12 and SYM10 antibodies. SMN was immunoprecipitated by Y12 and SYM10 antibodies, with a very small amount also immunoprecipitated by anti-coilin. SmB/B′ was immunoprecipitated by Y12 and SYM10 but not by anti-coilin. The symbol ’*’ denotes detection of antibodies used for immunoprecipitation.

To investigate directly the methylation of endogenous coilin in undifferentiated and differentiated SH-SY5Y cells, we carried out immunoprecipitation of sDMA-containing proteins using the antibodies Y12 and SYM10, both of which recognize sDMA motifs (24,34) (Figure 5). In both undifferentiated and RA+BDNF-differentiated SH-SY5Y cells, coilin is immunoprecipitated with both antibodies, demonstrating that the protein is methylated in differentiated and undifferentiated cells. The Y12 antibody appears to precipitate coilin slightly more efficiently in differentiated cells. However, this is not seen using the SYM10 antibody, which was raised specifically to recognize sDMA. Both Y12 and SYM10 also efficiently immunoprecipitate the Sm protein components of snRNPs, which are known to contain sDMA motifs. SMN is seen in the fractions immunoprecipitated with SYM10 and Y12. This is likely to be as a result of interaction between SMN and Sm proteins as SMN does not have an arginine motif predicted to be dimethylated (35). The ability of anti-sDMA antibodies to coimmunoprecipitate SMN has previously been observed in some, but not all, HeLa cell lines (35). Interestingly, immunoprecipitation of endogenous coilin (Figure 5A) does not result in the coimmunoprecipitaiton of Sm proteins in undifferentiated or differentiated cells. This suggests that only minor amounts of these proteins, if any, interact under these conditions. Likewise, the amount of SMN coimmunoprecipitated with coilin is barely detectable and is not increased in differentiated cells.

### Transient overexpression of SmB or coilin does not increase the colocalization of CBs and gems in neuroblastoma cells

**Figure 6:**
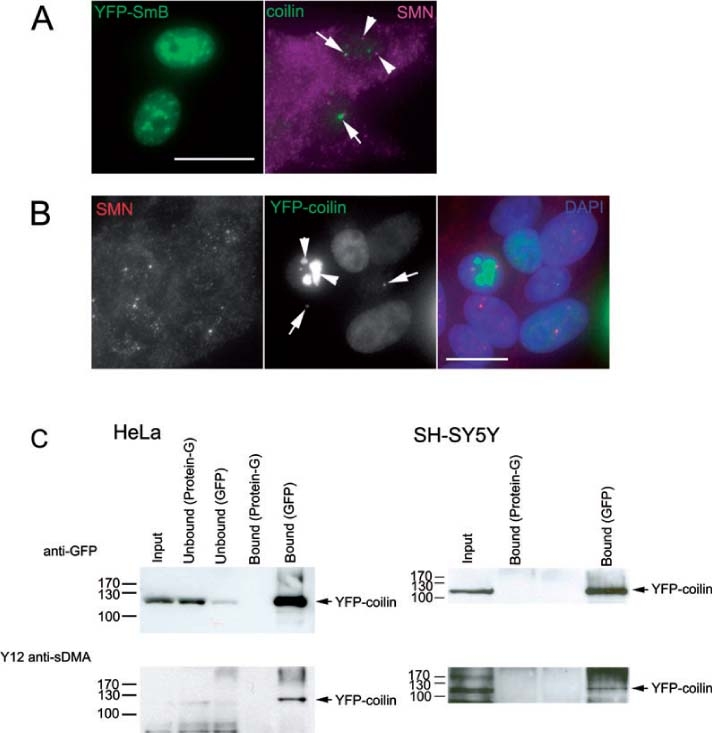
**Over-expression of SmB or coilin does not increase the colocalization of CBs and gems.** A) Expression of YFP-SmB in SH-SY5Y cells does not cause CBs and gems to colocalize. SH-SY5Y cells were transfected with a plasmid to express YFP-SmB (green in left hand panel). Detection of SMN (magenta) and coilin (green) in these cells reveals separate CBs (arrows) and gems (arrowheads). Bar = 10 μm. B) YFP-coilin shows aberrant localization in SH-SY5Y cells. Cells transfected with a plasmid to express YFP-coilin (centre panel and green in right hand panel) show diffuse nucleoplasmic signal for YFP-coilin in most cells, with discrete CBs (arrows) seen in only a few cells. Accumulation of YFP-coilin in the nucleolus is also commonly seen (arrowheads). Detection of SMN in these cells (left hand panel and red in right hand panel) shows no obvious disturbance of SMN distribution. Bar = 10 μm. C) YFP-coilin is methylated in HeLa and SH-SY5Y cells. YFP-coilin was immunoprecipitated from whole cell lysates using anti-GFP antibodies covalently bound to protein G–sepharose, separated by SDS–PAGE and blotted to nitrocellulose membrane. Anti-GFP (top panels) and Y12 anti-sDMA (bottom panels) were used to probe duplicate blots. Both antibodies detected a band representing YFP-coilin in both cell lines, confirming that YFP-coilin contains the symmetrical dimethylarginine motif in SH-SY5Y as well as in HeLa cells.

Previous studies in primary human fibroblasts demonstrated that expression of exogenous Sm protein was sufficient to induce the transient formation of CBs containing coilin, SMN and snRNPs (36). On-going snRNP biogenesis has recently been demonstrated to be essential for CB integrity (37). However, expression of yellow fluorescent protein (YFP)-tagged SmB in SH-SY5Y cells does not result in increased colocalization between the two nuclear bodies (Figure 6A). Cells expressing YFP-SmB contain both SMN and coilin in only 7% of total nuclear bodies (*n* = 67), which is comparable to untransfected cells (Figure 2).

The CB protein, coilin, has also been implicated in enabling the accumulation of SMN and snRNPs in CBs. Nuclei of cells from a coilin knockout mouse contain residual CBs that do not accumulate SMN or snRNPs (38), with a direct interaction between methylated coilin and SMN the likely molecular mechanism for the recruitment of SMN/snRNP complexes to CBs (25). Transient expression of YFP-coilin was, however, not well tolerated in SH-SY5Y cells (Figure 6B), with only a minor proportion of transfected cells showing YFP-coilin in CBs (arrows) while most cells displayed diffuse nucleoplasmic YFP-coilin, or accumulations of the tagged protein in nucleoli (arrowheads). To ensure that this aberrant localization was not caused by an inability of the cells to methylate YFP-coilin, anti-GFP antibodies were used to immunoprecipitate YFP-coilin from transfected SH-SY5Y cells and from a HeLa cell line constitutively expressing YFP-coilin in which wild-type localization is seen (27). The YFP-coilin immunoprecipiated from both cell lines was recognized by the anti-sDMA antibody, Y12 (Figure 6C), showing that YFP-coilin is methylated in SH-SY5Y cells as well as in HeLa cells.

### The level of SMN, but not that of coilin or Sm proteins, increases on differentiation

**Figure 7:**
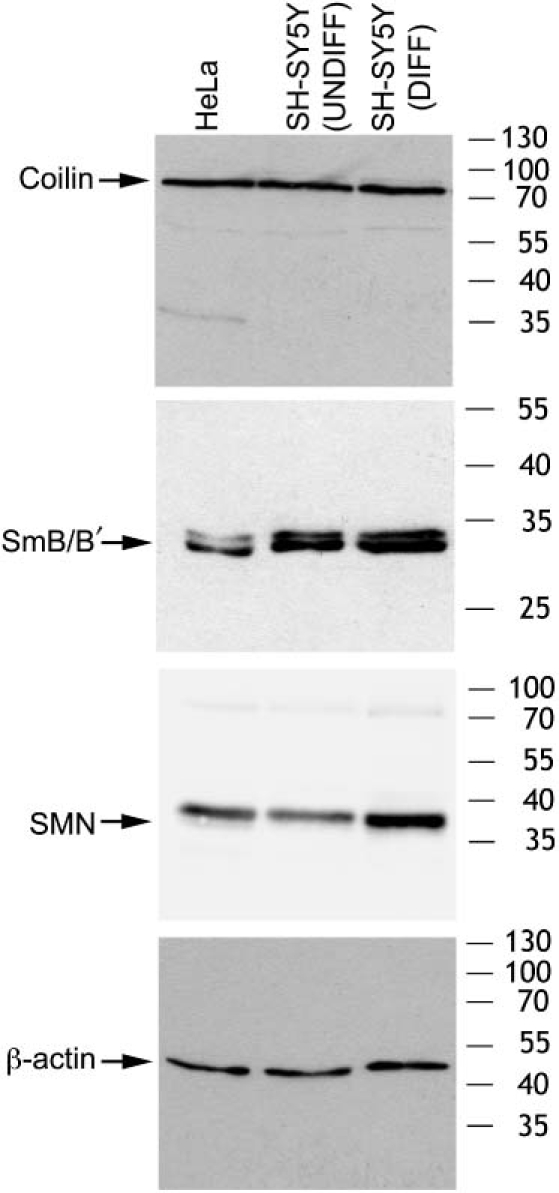
**SMN levels increase during differentiation of SH-SY5Y cells.** Equal amounts of whole cell lysates from HeLa cells, undifferentiated SH-SY5Y cells and BDNF-differentiated SH-SY5Y cells were separated by SDS–PAGE and blotted to nitrocellulose membrane. The amount of coilin was equal in all three samples. SmB/B′ was present in slightly lower amounts in HeLa cells than in SH-SY5Y cells and unaffected by differentiation of the cells. The level of SMN showed a marked increase (60%, normalized to β-actin levels) following differentiation of SH-SY5Y cells. β-actin is included as a loading control.

To investigate the potential changes in expression of endogenous nuclear body proteins associated with differentiation of SH-SY5Y cells, whole cell lysates of undifferentiated and BDNF-differentiated cells were separated on polyacrylamide gels alongside HeLa cell lysates and transferred to nitrocellulose membranes. Immunological detection of endogenous coilin, Sm proteins and SMN revealed a marked increase in SMN on differentiation (Figure 7). No increase or decrease was seen in the levels of coilin or Sm proteins.

### Overexpression of SMN is sufficient to cause complete colocalization of CBs and gems in SH-SY5Y cells

**Figure 8:**
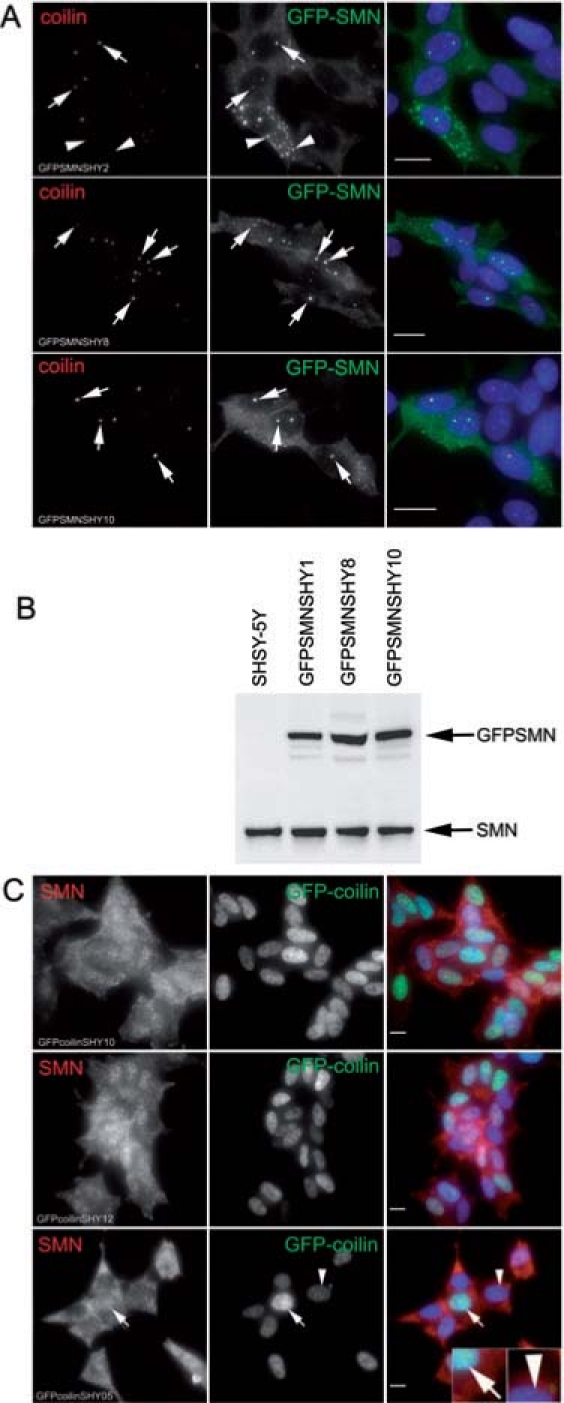
**Expression of GFP-SMN leads to complete colocalization of CBs and gems.** A) Three independent SH-SY5Y cell lines (clones SHY2, SHY8 and SHY10) expressing GFP-SMN show coilin, detected with anti-coilin antibodies (red on overlays) and GFP-SMN (green on overlays) fully colocalized within the nucleus into CB/gem complexes (arrows). A few cells show cytoplasmic accumulations of GFP-SMN (arrowheads). These do not contain coilin. Bar = 10 μm. B) Immunoblot of whole cell lysates from three independent SH-SY5Y cell lines detected with anti-SMN antibodies. Expression levels of the GFP-SMN fusion protein are approximately 75% (GFPSMNSHY1), 95% (GFPSMNSHY8) and 82% (GFPSMNSHY10) relative to endogenous SMN. C) Three independent SH-SY5Y lines (clones SHY10, SHY12 and SHY5) expressing GFP-coilin (green on overlays) localized diffusely throughout the nucleoplasm. SMN, detected with anti-SMN antibodies (red on overlays), also fails to localize to nuclear bodies in these cells. Line GFP-coilin-SHY5 (bottom row) shows a few small GFP-coilin-positive bodies. Some of these (arrow) also contain SMN, while some (arrowhead) do not. Bar = 10 μm.

Overexpression of SMN in HeLa cells has previously been demonstrated to result in cytoplasmic foci (26,27) and numerous small nuclear bodies. To investigate the effect of expressing excess SMN in undifferentiated SH-SY5Y cells, we established a number of cell lines stably expressing GFP-SMN (Figure 8A). In all of the cell lines examined, GFP-SMN was found in the cytoplasm and in a small number of nuclear bodies. These bodies resembled canonical CBs by size and number and were demonstrated to contain coilin. No nuclear bodies containing coilin alone or containing SMN alone (gems) were detected in these cell lines, suggesting that the amount of SMN present is the key determinant of nuclear body architecture in these cells. Analysis of the level of expression of GFP-SMN in these cell lines by western blot (Figure 8B) demonstrates an increase in total SMN levels in these lines of between 75 and 95%. To confirm that this effect was specific to the expression of exogenous SMN, we also established a number of cell lines expressing GFP-tagged coilin (Figure 8C). In agreement with results obtained from transient expression of YFP-coilin (Figure 6B), excess coilin is not well tolerated by SH-SY5Y cells, showing mostly diffuse localization within the nucleus. One cell line (GFP-coilin-SHY5) did show GFP-coilin-positive bodies in approximately 30% of nuclei. In this line, some of the coilin-positive bodies colocalize with endogenous SMN, but others do not, as is seen in the parental cell line (Figure 8C, inset). SMN localization is disrupted in these cell lines, with SMN-positive gems almost completely absent. This further emphasizes the importance of the balance between coilin and SMN levels for the integrity of nuclear CBs and gems.

### SMN depletion leads to a complete loss of Cajal bodies from SH-SY5Y cells

**Figure 9:**
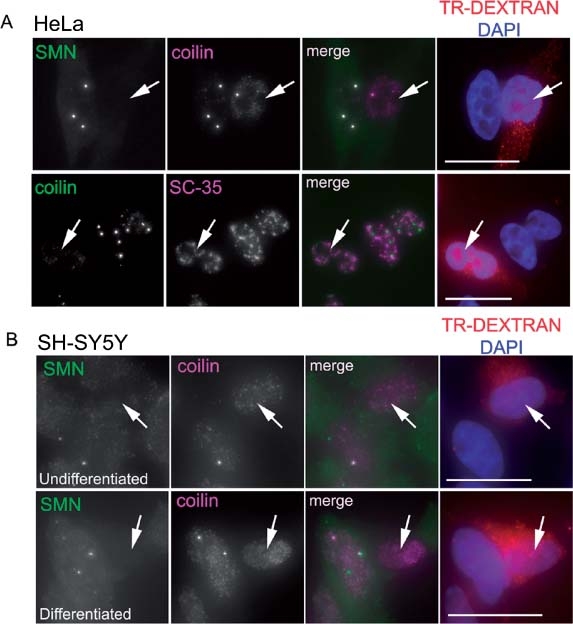
**Reduction of SMN expression with RNAi disrupts CBs as well as gems.** A) HeLa cells microinjected with siRNA duplexes to reduce SMN expression (demonstrated by red signal in TR-dextran panel, far right, and arrows) lack SMN-containing gems (left hand panel, green on overlays). Antibody detection of coilin (top row, magenta on overlays; bottom row, green on overlays) shows reduced CBs and increased diffuse nucleoplasmic signal. Antibody detection of the non-snRNP splicing factor, SC-35 (bottom row, magenta on overlays), reveals intact nuclear speckles. Bar = 10 μm. B) Undifferentiated (top row) and BDNF-differentiated (bottom row) SH-SY5Y cells microinjected with siRNA duplexes (red signal in TR-dextran panel, far right, and arrows) lack SMN-containing gems. Coilin-containing CBs (magenta on overlays) are also absent. Bar = 10 μm.

Depletion of SMN from HeLa cells using siRNA has been reported to disrupt CBs (28,33,39), resulting in small nuclear foci containing coilin and some accumulation of coilin in nucleoli. As CBs and gems represent the same structure in HeLa cells but are clearly separate in SH-SY5Y cells, we wanted to determine whether the reduced physical association between the two structures in SH-SY5Y cells reflects a reduced functional association. Cells were microinjected with siRNA duplexes to target SMN expression. The injected material was labelled with Texas Red dextran to allow precise identification of the experimental cells. In HeLa cells, we observed a general increase in diffuse nucleoplasmic coilin, together with a decrease in size and number of CBs (Figure 9A). The distribution of the SR splicing factor, SC-35, was not altered by SMN reduction, confirming that the rearrangements seen are specific to nuclear bodies and do not reflect a general disruption of nuclear architecture. In SH-SY5Y cells, either before or after differentiation, reduction of SMN led to a total loss of accumulation of coilin in CBs (Figure 9B), with only diffuse nucleoplasmic coilin observed. Thus, despite a less obvious relationship between CBs and gems in SH-SY5Y cells compared with HeLa cells, the loss of SMN has a greater effect on the integrity of CBs in these cells. This suggests that the functional relationship between CBs and gems is of at least equal importance in SH-SY5Y cells as in HeLa cells.

### Increased levels of SMN in SH-SY5Y cells lead to an increase in the rate of accumulation of Sm proteins in speckles

**Figure 10:**
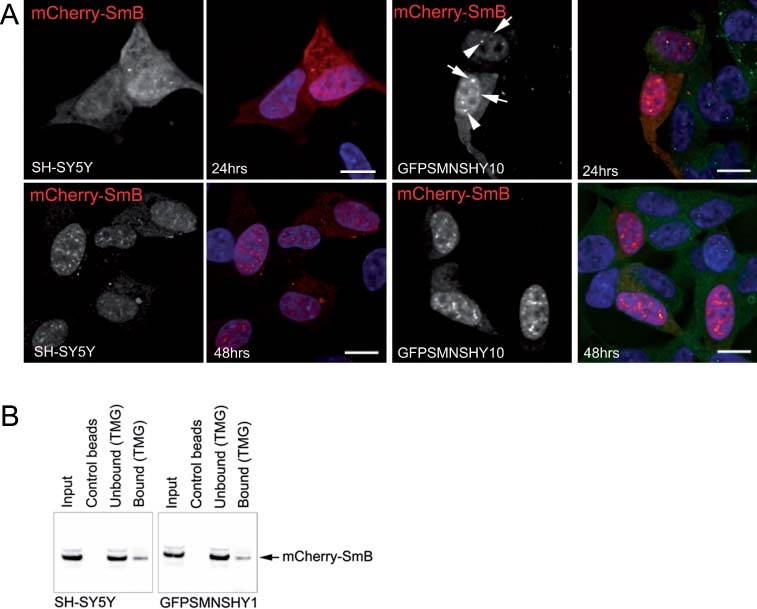
**Increased levels of SMN in SH-SY5Y cells lead to an increase in the rate of accumulation of Sm proteins in speckles.** A) Cells from lines SH-SY5Y and GFPSMNSHY1, expressing GFP-SMN, transiently transfected with a plasmid to express mCherry-SmB. After 24 h of expression (top row), the mCherry-SmB signal is seen throughout the cell in SH-SY5Y cells, while cells from line GFPSMNSHY1 show clear accumulation of mCherry-SmB in nuclear speckles (arrows) and CBs (arrowheads). After 48 h of expression (bottom row), both cell lines show a similar accumulation of mCherry-SmB in nuclear speckles. Bar = 10 μm. B) Immunoisolation of U snRNPs from cells expressing mCherry-SmB for 24 h using antibodies to the TMG cap of U snRNAs. Similar amounts of mCherry-SmB are associated with U snRNAs from the two cell lines [compare bound (TMG) to unbound (TMG)].

The uptake of newly made snRNPs into the nucleus can be followed using fluorescently tagged Sm proteins (7). In cell lines studied previously, newly made snRNPs are at first seen diffusely within the cell, and localize to CBs prior to their eventual accumulation in nuclear speckles. Important modifications of the snRNA core of snRNPs are carried out in the CB (8,9). Interfering with snRNP biogenesis using metabolic inhibitors (27) or reduction of SMN levels (39) has been demonstrated to prevent or reduce the accumulation of newly made snRNPs in speckles. To determine the effect of increased levels of SMN on the efficiency of snRNP maturation in SH-SY5Y cells, the parental cell line and a cell line constitutively expressing GFPSMN (GFPSMNSHY10) were transfected with a plasmid to express the core snRNP protein, SmB tagged with mCherry (40). Cells were fixed after 24 and 48 h and the distribution of mCherry-SmB determined (Figure 10). In SH-SY5Y cells expressing only endogenous SMN protein, mCherry-SmB is largely diffuse within the cell after 24 h of expression. In contrast, cells of line GFPSMNSHY10, which overexpress SMN by approximately 75%, already show clear accumulation of mCherry-SmB in nuclear speckles (arrows) and CBs (arrowheads) after 24 h of expression. After 48 h of expression, mCherry-SmB shows a clear pattern of speckles within the nucleus in both cell lines. To determine whether this increase in the rate of accumulation of snRNPs in speckles results from an increased efficiency of SMN-mediated snRNP assembly in the cytoplasm or from an increased efficiency of stages of snRNP maturation carried out in the CB, we isolated U snRNAs from the mCherry-SmB-transfected cells using antibodies against the characteristic trimethylguanosine (TMG) 5′ cap structure. Analysis of the bound and unbound fractions using antibodies to mCherry revealed a similar proportion of mCherry-SmB assembled onto snRNAs at the 24 h time-point in both cell lines. This suggests that the increased efficiency with which cells expressing GFP-SMN accumulate mCherry-SmB in speckles results from an increased efficiency of nuclear stages of snRNP maturation and/or transport, rather than an increase in the rate of assembly of Sm proteins onto snRNAs by SMN in the cytoplasm.

## Discussion

The sequential use of retinoic acid and BDNF can reproducibly differentiate SH-SY5Y human neuroblastoma cells into cells of neural appearance with extensive neurite outgrowth. A comparison of nuclear bodies containing SMN (gems) and coilin (CBs) reveals these two structures to be largely separate in undifferentiated cells (10% of nuclear bodies contain both antigens). Following differentiation, the percentage of gems and CBs colocalized into a CB/gem complex increases reproducibly to approximately 25%. Detailed comparison of adult and foetal tissues has demonstrated an increase in colocalization of coilin and SMN during development in many tissue types (3,29). This suggests that increased colocalization of CBs and gems is a feature of cellular differentiation *in vivo*. Studies using the rat PC12-derived cell line U61 (41) also reported an increased recruitment of SMN to nuclear CBs during neuritogenesis, although an increased number of gems devoid of coilin was also seen in differentiated cells. It seems likely, therefore, that increased presence of SMN in CBs is characteristic of increased cellular differentiation *in vitro* and maturation of tissues *in vivo*.

Methylation of proteins, particularly the symmetrical dimethylation of arginine motifs in coilin, has been implicated in regulation of nuclear body formation (24,25). Using myc-tagged coilin expressed in HeLa cells, Matera et al. demonstrated that methylated coilin binds SMN with a higher efficiency than does unmethylated coilin. Certain strains of HeLa in which CBs and gems fail to colocalize do not methylate GFP-coilin efficiently. Inhibition of protein methylation in SH-SY5Y cells using AdOx or MTA results in a decreased association between CBs and gems in undifferentiated and in differentiated cells (Figure 3). This is associated with a general decrease in coilin-positive bodies. These results are in partial agreement with the previous results in HeLa cells (24,25), although in HeLa cells MTA, but not AdOx, caused dissociation of CBs and gems. This difference suggests that the regulation of nuclear body morphology may be subtly different in neural cells compared with other cell types. Reduction in expression of PRMT5, responsible for the addition of methyl groups to coilin, results in a severe disruption of CBs in HeLa cells [this study and (33)], but has a very little effect on nuclear bodies in SH-SY5Y cells. Despite the disruption seen in HeLa cells, coilin and SMN remain closely colocalized. This suggests that the direct molecular interactions between methylated coilin and SMN may not be the key determinant of whether CBs and gems colocalize. Symmetrical arginine dimethylation of core snRNP, Sm, proteins is required for their efficient assembly onto snRNAs during the cytoplasmic stages of snRNP assembly (42–44), as SMN binds preferentially to methylated Sm proteins. Downregulation of the methyltransferases PRMT5 and PRMT7 has been shown to decrease the efficiency of snRNP assembly (33). As disruption of CBs has previously been seen following inhibition of snRNP biogenesis (36,37,45), reduction of snRNP maturation may contribute to the separation of gems and CBs in cells treated with global methylase inhibitors.

The prevailing view is that the purpose of accumulation of factors into the CB/gem complex is to provide increased local concentrations of rate-limiting factors for assembly and recycling pathways and so to increase their efficiency (11,46). Cultured primary cells remain viable in the absence of visible CBs (47), while the coilin knockout mouse shows somewhat decreased viability depending on the genetic background (38). These data suggest that processes normally occurring in CBs can be carried out elsewhere within the nucleus, but probably less efficiently. As increasing information becomes available about the effects of overexpression and reduction of expression of proteins involved in the CB/gem complex, it is clear that the balance between these molecular components is key to the correct formation and, by inference, function of the complex. Cells from the coilin knockout mouse contain residual CBs that accumulate nucleolar markers typical of canonical CBs but do not recruit SMN or snRNPs. In these cells, SMN is found in gems, separate from other CB markers (38). Expression of exogenous coilin in these cells leads to the formation of normal CB/gem complexes containing coilin, SMN and Sm proteins. Moderate overexpression of coilin in HeLa cells or primary cells does not lead to increased number of CBs (27,48–50), with tagged versions of coilin showing wild-type localization. However, high levels of expression of GFP-coilin in HeLa cells can lead to disruption of the CB/gem complex (51). Disruption of CBs is seen in SH-SY5Y cells transiently expressing exogenous coilin, with very few transfected cells showing correct localization of YFP-coilin (Figure 6). Stable overexpression of GFP-coilin results in an almost complete loss of CBs and gems (Figure 8C). Expression of GFP-tagged Sm proteins, predicted to result in upregulation of the snRNP maturation pathway, is sufficient to cause the formation of CBs containing coilin, SMN and Sm proteins in primary cell lines that normally lack CBs. Furthermore, GFP-Sm expression causes the colocalization of CBs and gems in strains of HeLa cell where they are usually separate (36). This suggests that, in these cells, the CB/gem complex forms in response to upregulation of snRNP maturation. In SH-SY5Y cells, however, expression of YFP-SmB has no effect on nuclear bodies (Figure 4B), raising the possibility that there is a difference in the regulation of nuclear body formation between neural SH-SY5Y cells and other cell types studied to date. If this proves to be the case for other neural cell types, it may be highly significant for elucidating the molecular defects responsible for SMA.

The most striking differentiation-associated change in endogenous nuclear body proteins in neural cells is an increase in SMN (Figure 7). During differentiation in two neural cell types, human SH-SY5Y (this study) and rat U61 (41), SMN increases its level and its accumulation in CBs. While this does not rule out upregulation of SMN as a general feature of cellular differentiation, it does suggest that increased SMN levels are associated with neural differentiation and the extension of neurites. Cells unable to sufficiently increase their SMN levels may differentiate less efficiently and be susceptible to damage or death. Expression of GFP-SMN in SH-SY5Y cells leads to complete colocalization of coilin and SMN into CB/gem complexes (Figure 8). Thus, in these cells, it is not a lack of coilin, defects in coilin methylation or low levels of snRNP maturation that lead to the separation of CBs and gems, but a restrictive level of SMN. The complete absence of nuclear gems and CBs in SH-SY5Y cells with SMN expression lowered by RNAi (Figure 9) supports this view. Expression of additional SMN in SH-SY5Y cells not only causes the colocalization of coilin and SMN into a CB/gem complex but also increases the rate of accumulation of newly imported snRNPs in nuclear speckles (Figure 10). This observation ties the presence of a CB/gem complex containing both coilin and SMN to an increased efficiency of snRNP maturation.

It still remains to be discovered whether defects in snRNP biogenesis are responsible for the motor neuron-specific pathology seen in SMA. Disturbance of snRNP maturation has been demonstrated in a severe mouse model of SMA (22,23) and in cells with levels of SMN reduced to 5% of wild type (23). Associated tissue-specific defects in pre-mRNA splicing were seen. Defects in motor axon outgrowth and path finding have been documented in zebra fish models of SMN (15,16). However, these were rescued by purified snRNPs (16), suggesting a direct link between the function of SMN in snRNP maturation and the cytoskeletal phenotype in axons and growth cones. This, again, suggests that SMN is involved in delicately balanced systems: the reduction of overall levels of SMN may cause a preferential loss of axon-specific functions of SMN in order to preserve the essential housekeeping role of SMN in supplying sufficient snRNPs to the splicing machinery. More drastic or more prolonged lack of SMN may lead to disruption of snRNP biogenesis and splicing.

## Materials and Methods

### Cell culture and differentiation

SH-SY5Y and HeLa cell lines were routinely cultured in Dulbecco's modified Eagles' medium (DMEM, Invitrogen) supplemented with 10% foetal bovine serum (Invitrogen). For immunofluorescence assays, cells were grown on coverslips and transfected (if necessary) using Effectene transfection reagent (Qiagen) according to the manufacturer's instructions. For the preparation of cell lysates, cells were grown in 10 cm diameter dishes. To obtain differentiated cultures of SH-SY5Y cells, retinoic acid (Sigma) was added to the medium at a final concentration of 10 nM for 10 days. Alternatively, retinoic acid was added to the medium for 4 days, after which the medium was replaced with serum-free medium containing 2 nM BDNF (Sigma). The cells were cultured for a further 6 days. To inhibit protein methylation, cells were cultured for 24 h with either 750 μM MTA (Sigma) or 100 μM AdOx (adenosine periodate, Sigma).

### Cell fixation, immunostaining and microscopy

HeLa cells grown on glass coverslips were fixed for 5 min at room temperature with 3.7% paraformaldehyde in PHEM buffer [60 mM PIPES, 25 mM HEPES, 10 mM EGTA, 2 mM MgCl_2_ (pH 6.9)]. Immunostaining was carried out as described previously (27). Cells were mounted in Vectashield medium (Vector labs). Antibodies used were rabbit 204/10 anti-coilin (a gift from A. I. Lamond) (dilution 1:500), mouse monoclonal antibody (mAb) MANSMA1 anti-SMN (29) (dilution 1:10), SC-35 (Sigma) (dilution 1:2000), fluorescein isothiocyanate (FITC)- and tetramethylrhodamine isothiocyanate

(TRITC)-conjugated goat anti-mouse, Cy5- and TRITC-conjugated goat anti-rabbit immunoglobulin (IgG) (Jackson ImmunoResearch Laboratories) (dilution 1:250). Immunostained specimens were examined using a Zeiss Axiovert 2 (63× objective) and recorded using a Hammamatsu C4742-80-12AG camera with Volocity software (Improvision) (Figures 1 and 2) or examined and recorded using a DeltaVision Spectris Deconvolution microscope (60× or 100× objective) (Figures 4, 6, 8, 9 and 10). In both cases, optical sections separated by 200 nm were collected using a binning of 2 × 2.

### Image processing and analysis

Maximum intensity projections of serial sections were generated using Volocity (Improvision) and colour overlays generated, where appropriate, using Adobe Photoshop. Counts of number of nuclear bodies were made using Volocity (Improvision) and statistical analyses performed using Prism (GraphPad).

### siRNA knockdowns

For knockdown of PRMT5 by electroporation, cells were electroporated with SmartPool siRNA duplexes (Dharmacon) using an AMAXA Nucleofector program G-04, 25 μg siRNA per 2 × 10^6^ cells, with Ingenio electroporation solution (Mirus) according to the manufacturer's instructions. Sequences used were PRMT5: CAACAGAGAUCCUAUGAUU (A), CCAAGUGACCGUAGUCUCA (B), UGGCACAACUUCCGGACUU (C), CGAAAUAGCUGACACACUA (D) or a mixture of all four, SMN: CAGUGGAAAGUUGGGGACA, negative control targeting luciferase: UAAGGCUAUGAAGAGAUAC, positive control targeting cyclophilin B: GGAAAGACUGUUCCAAAAA. For knockdown of SMN by microinjection, cells were microinjected with siRNA duplexes diluted in injection buffer [100 mM glutamic acid pH 7.2 (with citric acid); 140 mM KOH; 1 mM MgSO_4_ and 1 mM DTT] containing lysine-fixable Texas Red-labelled dextran (Invitrogen) using an Eppendorf Injectman NI 2 mounted on a Zeiss Axiovert 40 microscope.

### Plasmid constructs and cell lines

Plasmids to express EGFP-SMN, EGFP-coilin, EYFP-SmB and EYFP-coilin have been described previously (27,36). The plasmid to express mCherry-SmB was generated by subcloning the SmB cDNA from pEYFP-SmB into plasmid pmCherry-C1 (40). Stable cell lines were established using G418 selection of SH-SY5Y cells following transfection with Effectene transfection reagent (Qiagen) as described previously (36).

### Preparation of cell lysates, immunoblotting and immunoprecipitation

Lysates were prepared as described previously (27,36), electrophoresed on a 10% SDS polyacrylamide gel and transferred to nitrocellulose membranes for immunoblotting. Antibodies used were mAbs Y12 anti-sDMA (Abcam, 1:500); MANSMA1 anti-SMA (a gift from Professor G. Morris, 1:500); p∂ anti-coilin (Sigma, 1:1000), anti-α-tubulin (Sigma, 1:2000) and AC-15 anti-β-actin (Sigma, 1:2000); rabbit polyclonals 204/10 anti-coilin (a gift from Professor A. I. Lamond, 1:500) and anti-PRMT5 (Abcam, 1:250). Secondary antibodies were horseradish peroxidase (HRP)-conjugated anti-mouse or anti-rabbit (Pierce, 1:20 000). Detection was carried out with ECL Plus Western Blotting Detection System (GE Healthcare) and imaged using a Fujifilm LAS-3000 imaging system. Immunoprecipitation using anti-GFP antibodies (Roche) was performed as described previously (52). Primary antibodies used were mAb anti-GFP (Roche, 1:1000) and Y12 anti-sDMA (Abcam). To immunoprecipitate endogenous proteins, 500 μg of pre-cleared whole cell lysate was shaken overnight at 4°C with 5 μg of Y12 (Abcam) or SYM10 (Upstate Biotech) anti-sDMA antibodies or with a mixture of 12 μg p∂ anti-coilin (Sigma) and 2 μg of anti-coilin (BD Biosciences) (53). Protein G–Sepharose beads (50 μL per sample) were then added and the samples shaken at 4°C for a further 4 h. Following four brief washes with cell lysis buffer, the beads were resuspended in SDS–PAGE sample buffer for electrophoresis and subsequent immunodetection. Immunoprecipitation of snRNPs using anti-trimethylguanosine antibodies (Calbiochem) was carried out as described previously (36). Antibodies for detection were rat monoclonal to dsRed (a gift from Professor H. Leonhardt, 1:150) and HRP-conjugated anti-rat (Pierce, 1:20 000).
